# Supplementary material for: Data Resource Profile: The Multiple Sclerosis Documentation System 3D and AOK PLUS Linked Database (MSDS-AOK PLUS)
Source: J Clin Med. 2023 Feb 10;12(4):1441. doi: 10.3390/jcm12041441 (PMC9962623; doi:10.3390/jcm12041441)
Supplement: Supplementary file 1 [file jcm-12-01441-s001.zip › jcm-2193419-supplementary.pdf]

Supplementary Material

Data Resource Profile: The Multiple Sclerosis Documentation System 3D and AOK PLUS Linked Database (MSDS-AOK PLUS)

Marco Ghiani [1], Evi Zhuleku [2,*], Anja Dillenseger [3], Ulf Maywald [4], Andreas Fuchs [4], Thomas Wilke [1], and Tjalf Ziemssen [3]

[1] Institut für Pharmakoökonomie und Arzneimittellogistik e.V. (IPAM e.V.), 23966 Wismar, Germany
[2] Cytel Inc., 10785 Berlin, Germany
[3] ZKN, Zentrum für Klinische Neurowissenschaften, Neurologische Klinik und Poliklinik für Neurologie, Universitätsklinikum Carl Gustav Carus, 01307 Dresden, Germany
[4] AOK PLUS, 01067 Dresden, Germany
* Correspondence: evi.zhuleku@cytel.com; Tel.: +49-301-6637-5695

Table S1. Components used to compute Charlson Comorbidity Index (CCI).

No	Comorbidity	Charlson Score	ICD-10 Code
1	Coronary artery disease	1	I20.-, I21.-, I22.-, I23.-, I24.-, I25.-
2	Congestive heart failure	1	I11.-, I50.-
3	Peripheral vascular disease	1	I73.-, I74.-, I77.-
4	Cerebrovascular disease	1	G45.-, G46.-, I6.-
5	Dementia	1	F00.-, F01.-, F02.-, F03.-, G30.-
6	Chronic pulmonary disease	1	J4.-, J6.- w/o J67.-, J68.-, J69.-
7	Connective tissue disorder	1	M05.-, M06.-, M07.-, M08.-, M3.-
8	Peptic ulcer disease	1	K25.-, K26.-, K27.-, K28.-
9	Mild liver disease	1	B18.-, K70.-, K73.-, K75.-
10	Diabetes mellitus without complications	1	E109.-, E119.-, E129.-, E139.-, E149.-
11	Hemiplegia	2	G81.-, G82.-
12	Moderate or severe renal disease	2	N17.-, N18.-, N19.-
13	Diabetes mellitus with end-organ damage	2	E10.-, E11.-, E12.-, E13.-, E14.- w/o [No 10]
14	Tumor without metastases, leukemia, lymphoma, multiple myeloma	2	C% w/o [No 16]
15	Moderate or severe liver disease	3	K72.-, K74.-, I85.-
16	Metastatic solid tumor	6	C77.-, C78.-, C79.-, C80.-
17	AIDS	6	B20.-, B21.-, B22.-, B23.-, B24.-
18	Age factor (was excluded from index)	For each decade ≥50 years of age, 1 point was added to the score	

AIDS, Acquired Immunodeficiency Syndrome
